# A Structurally and Functionally Biomimetic Biphasic Scaffold for Intervertebral Disc Tissue Engineering

**DOI:** 10.1371/journal.pone.0131827

**Published:** 2015-06-26

**Authors:** Andrew Tsz Hang Choy, Barbara Pui Chan

**Affiliations:** Tissue Engineering Laboratory, Department of Mechanical Engineering, The University of Hong Kong, Hong Kong Special Administrative Region, China; University of Pennsylvania, UNITED STATES

## Abstract

Tissue engineering offers high hopes for the treatment of intervertebral disc (IVD) degeneration. Whereas scaffolds of the disc nucleus and annulus have been extensively studied, a truly biomimetic and mechanically functional biphasic scaffold using naturally occurring extracellular matrix is yet to be developed. Here, a biphasic scaffold was fabricated with collagen and glycosaminoglycans (GAGs), two of the most abundant extracellular matrix components in the IVD. Following fabrication, the scaffold was characterized and benchmarked against native disc. The biphasic scaffold was composed of a collagen-GAG co-precipitate making up the nucleus pulposus-like core, and this was encapsulated in multiple lamellae of photochemically crosslinked collagen membranes comprising the annulus fibrosus-like lamellae. On mechanical testing, the height of our engineered disc recovered by ~82-89% in an annulus-independent manner, when compared with the 99% recovery exhibited by native disc. The annulus-independent nature of disc height recovery suggests that the fluid replacement function of the engineered nucleus pulposus core might mimic this hitherto unique feature of native disc. Biphasic scaffolds comprised of 10 annulus fibrosus-like lamellae had the best overall mechanical performance among the various designs owing to their similarity to native disc in most aspects, including elastic compliance during creep and recovery, and viscous compliance during recovery. However, the dynamic mechanical performance (including dynamic stiffness and damping factor) of all the biphasic scaffolds was similar to that of the native discs. This study contributes to the rationalized design and development of a biomimetic and mechanically viable biphasic scaffold for IVD tissue engineering.

## Introduction

In developed countries, lower back pain occurs in more than 70% of people, affecting their quality of live [1]. Although the patho-etiology of degenerative disc disease is still unknown [2], the physiological changes that take place at the cellular level as an intervertebral disc (IVD) degenerates are well documented. Young and healthy IVD are biphasic in structure. They are comprised of an inner core, or nucleus pulposus (NP), which is rich in glycosaminoglycans (GAGs) that attract water, and in this way, resist deformation against compressive loads and recover lost disc height upon load reduction through swelling [2–4]. The NP core is surrounded by the annulus fibrosus (AF), which is comprised of 15–40 lamellae, which are concentric, interwoven layers composed primarily of collagen. The outer AF has distinctive layer structure while the inner AF has higher GAG content transiting to NP. The lamellae confine the NP and thus prevent any lateral deformation due to compressive loads [2]. The biphasic structure of the disc works to maintain the disc height during variations in diurnal loading [2, 5]. The cells are round and chondrocyte-like in the NP and elongated and fibroblast-like in the AF [2]. Disc degeneration is characterized by a reduced GAG content and herniation of the NP due to a weakened AF, and these together gradually result in a reduced disc height [2].

Even when the cause of lower back pain cannot be confirmed, treatment of the disc degeneration may be important for resolving the pain [2, 6]. Currently, the only intervention for a degenerated disc, especially a severely damaged one, is surgery [6]. However, surgical procedures can also be problematic; for example, removal of a degenerated disc by discectomy renders the joint unstable [6]. Spinal fusion involves fusing adjacent vertebral bones by insertion of an interbody cage and is considered to be the clinical gold standard for treating degenerative disc disease. However, while this procedure stabilizes the joint, it compromises movement, alters the biomechanics of the vertebral column and risks the propagation of disc degeneration to other discs [6]. Prosthetic disc replacement offers to preserve the joint with adequate mechanical strength and hence movement, but there are also limitations with this procedure, such as subsidence and implant aging, which might require additional surgery [6].

Tissue engineering presents a potential biological solution for replacing the structure and function of a degenerative disc. This is particularly relevant at late stages of degeneration when a biocompatible scaffold is produced to provide temporary mechanical support as well as the structural features required for remodeling and maintenance of the engineered tissue by viable cells. Investigations into the use of biomaterials such as alginate, collagen, hyaluronan and collagen/GAG as a possible NP replacements, and polyglycolic acid/polylactic acid, silk and alginate/chitosan fibers as possible AF replacements have shown promising results [7, 8]. Recent research has focused on mimicking the biphasic structure of the native AF and NP [9–14]. Most of these studies demonstrated that whereas the cells cultured in biphasic scaffolds maintained a phenotype similar to their native counterparts, the corresponding matrix (such as GAG and type II collagen in the NP), accumulated but was inadequate. In one study by Bowles et al., engineered biphasic discs created from collagen (AF) / alginate (NP) were implanted to rat tails and showed similar GAG and hydroxyproline content to the native disc over six months. Their biphasic disc also demonstrated similar mechanical properties, such as equilibrium modulus, hydraulic permeability, and percent hysteresis comparable to the rat caudal disc [9], demonstrating the possibility to bioengineer disc replacements with mechanical viability.

To mimic the structure and function of native disc, an ideal biphasic scaffold should be made from biomaterials with excellent biocompatibility and with comparable mechanical properties of the native discs. Specifically, the structure should be composed of lamellae structures with comparable strength to the AF, and which encapsulate a nucleus that closely approximates the native NP. Collagen is a natural protein that exists in abundance in mammals, and which has low antigenicity and is thus a common biomaterial for scaffolds. To make the collagen stronger for the AF scaffold and more GAG-rich for the NP core, photochemically-crosslinked collagen membrane (PCM) [15] and collagen-GAG (CG) modifications [16] were developed by our group, respectively. The PCM was shown to have a tangential modulus of 20 MPa at 90% rupture strain, which is considerably stronger than that of non-crosslinked collagen membrane (3 MPa), and is comparable to the tensile modulus of an AF lamella in the circumferential direction, which is also 20 MPa [15]. Photochemical crosslinking involves exciting rose Bengal, a non-toxic photosensitizer with an argon laser at 514 nm. The crosslinking process is non-thermal and simple to control, and it may be integrated with the lamination process during construction of the biphasic scaffold. Furthermore, the GAG content was normalized against the level of hydroxyproline (HYP), which represents collagen, and the ratio used indicates the relative abundance of GAGs in GAG-rich tissues. Early reports suggested that, with the crosslinking methods available at the time, the GAG:HYP ratio could not exceed 2.5 [17], which is similar to that demonstrated in articular cartilage [3]. On the other hand, the chemically modified CG co-precipitation we developed [16] has improved the GAG:HYP ratio to >4.5, which is comparable to that in aged NP [3], although it is still much lower than that of juvenile NP [3].

In this work, we report the design of a new biphasic scaffold, made from natural biomaterial collagen and GAG using a repeated lamination method. We hypothesize that the biphasic scaffold will structurally and functionally mimic native disc, and that the number of layers of AF lamellae used in the scaffold is an important factor, which affects its functional properties. Specifically, we describe fabrication of a biphasic scaffold using CG and PCM. We also evaluate the histological, structural and functional parameters of the scaffold by benchmarking with native disc, and investigate the layering effect of the AF lamellae.

## Materials and Methods

### Preparation of the NP-like CG core

The NP-like CG core was fabricated and used as the nucleus of the biphasic construct, as previously described [16]. In brief, acid soluble collagen solution (354236, BD Biosciences, San Jose, CA, USA), ethylenediamine solution (EDA) (E26266, Sigma-Aldrich, USA) and 1-ethyl-3(3-dimethylaminopropyl) carbodiimide (EDC) (E7750, Sigma-Aldrich) were mixed to give a final concentrations of 0.037% collagen, 2.2M EDA and 0.044M EDC. The amounts of EDA and EDC corresponded to 5000 and 300 molar multiples of the carboxyl groups on collagen, respectively. The solution was mixed at room temperature on a sideway shaker at 30 rpm overnight during which time the collagen became aminated. Aminated collagen was then retrieved by dialysis against 0.02 M acetic acid at room temperature for 6 h, and then at 4°C for 2 days. Aminated collagen and chondroitin-6-sulphate (C4384, Sigma-Aldrich) at 0.4% (w/v) were mixed at a ratio of 1:1.5 (w/w) and centrifuged at the maximum speed (~3000 rpm) of a vortex-mixer (Thermo Scientific, MA, USA) to produce insoluble CG, which was retrieved by centrifugation at 16,100 g for 2 min (5415D, Eppendorf, Hamburg, Germany). The collected CG was shaped with a cylindrical mould of 5 mm diameter, and air dried into a cylindrical block for the next step (i.e., layering with PCM) of fabrication of the biphasic disc scaffold.

### Fabrication of the biphasic constructs

**Fig 1:**
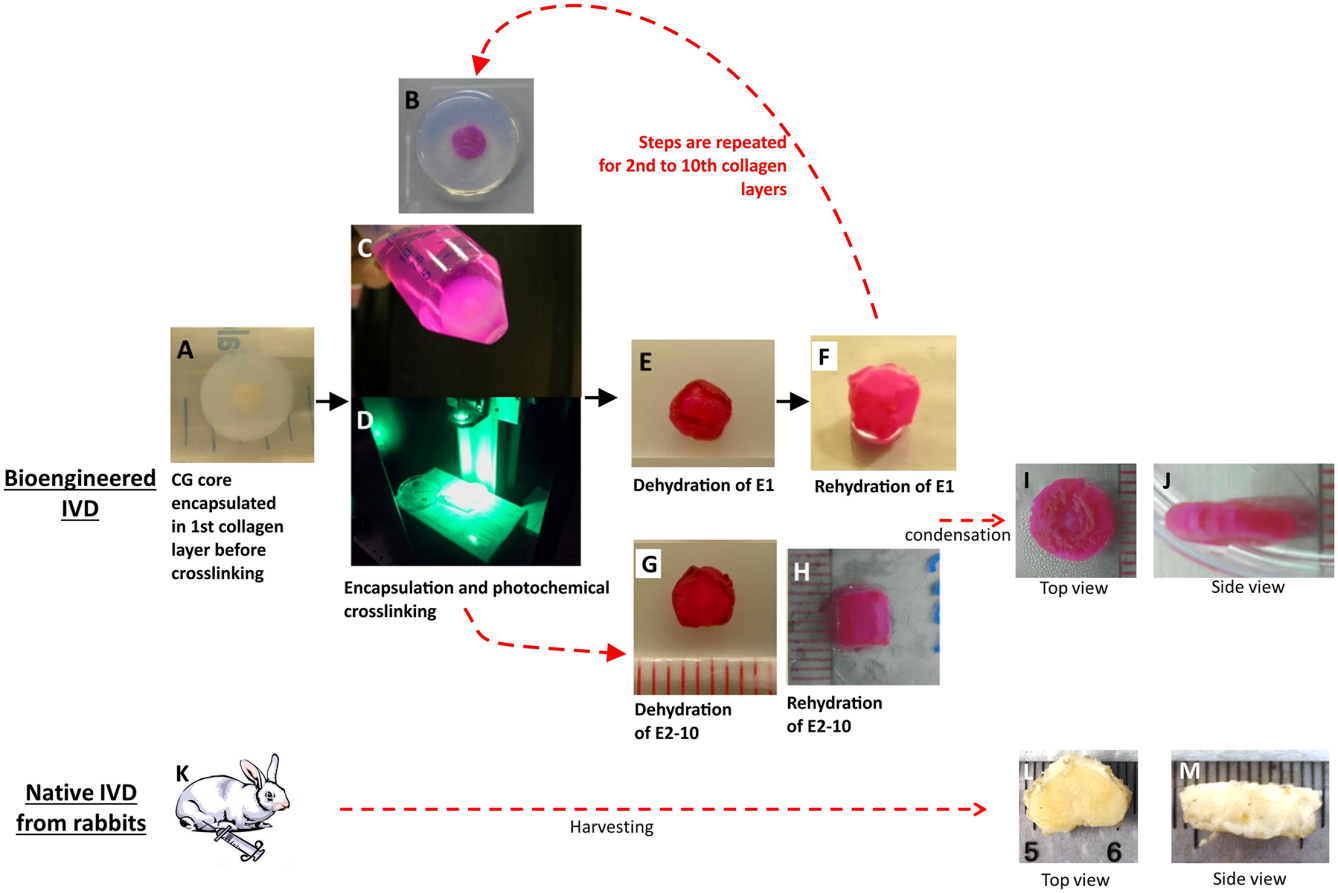
Series of images to show the fabrication of the biphasic scaffold an intervertebral disc. (A) A CG core was encapsulated in the first collagen layer before crosslinking. (B) Some of the photochemically-crosslinked CG cores were then encapsulated in a further 1 to 9 layers of collagen. (C) Immersion of the CG core with collagen layers in the photosensitizer, rose Bengal. (D) Irradiation of the construct with an argon laser at 514 nm for photochemical crosslinking. (E) Dehydration of the CG containing the 1^st^ collagen layer by rolling it on absorbent filter paper. (F) Dehydration of the CG core now encapsulated in the 2^nd^ to 10^th^ collagen layers. (G) The rehydrated CG core in one of the AF-like collagen lamella (E1). (H) Rehydrated CG core in two to ten collagen lamellae (E2 to E10). (I) Condensed biphasic scaffold in top view. (J) Condensed biphasic scaffold in side view. (K) Rabbit disc harvest. (L) Native rabbit disc in top view. (M) Native rabbit disc in side view.

The biphasic disc scaffold was fabricated by repeatedly laminating the NP-like CG core with photochemically crosslinked collagen lamellae to mimic the AF, as shown in Fig 1. The photochemical crosslinking procedure employed was a modified version of the one we previously reported for fabricating collagen membranes [15]. In brief, at 4°C acid soluble collagen solution was neutralized with sodium hydroxide and mixed with phosphate buffered saline (PBS) at pH 7.4 to prepare a 0.3% collagen gelling solution (w/v). One ml of the mixture was then transferred to a cylindrical mould of 16 mm diameter for gelation. At the start of gelation, the bottom of the mould was immersed in PBS pre-warmed to 37°C, and the top of the mould was in contact with a chilled plate (~4°C) for 5 min. This temperature difference between the bottom and top of the mould allowed just enough time for the dehydrated CG to be poured before it solidified. Further incubation of the whole mould at 37°C for 30 min resulted in the CG being encapsulated in a collagen hydrogel of 16 mm diameter and 5 mm tall (Fig 1A). This CG-gel composite was soaked in rose Bengal solution (R3877, Sigma) at a concentration of 0.001% (w/v) overnight (Fig 1C), and photochemically crosslinked by irradiating with an argon laser (Innova 300C, Coherent, California, USA; 514 nm) for 125 sec from the top to the bottom at a power of 200 mW (fluence = 25 J/cm^2^) (Fig 1D). The crosslinked CG-gel composite was dehydrated in a controllable manner by rolling on a piece of filter paper, until any loosely bound water in the collagen gel was removed (Fig 1E). The dehydrated gel of the composite thus made up one lamella (E1) of photochemically crosslinked collagen membrane surrounding the nuclear CG. (Fig 1G) This lamination process was repeated by encapsulating E1 in the 2^nd^ collagen layer (Fig 1B), and the process was repeated until eventually, biphasic constructs containing 1, 2, 4 or 10 lamella(e) were obtained (Fig 1H). As a finishing step to further condense the scaffold structure into a dense fibrous meshwork with appropriate dimensions and to bring the construct to similar loaded physiologically conditions, biphasic constructs, right before mechanically tested, were submitted to a pre-load, comprising of 150 sec at -0.6 MPa and then 600 sec at -0.1 MPa. These stress values correspond to the pressure in a normal human disc, such that the lower stress value was measured during lying down, and the higher stress value was measured during standing [18].

### Biomechanical Characterization

**Fig 2:**
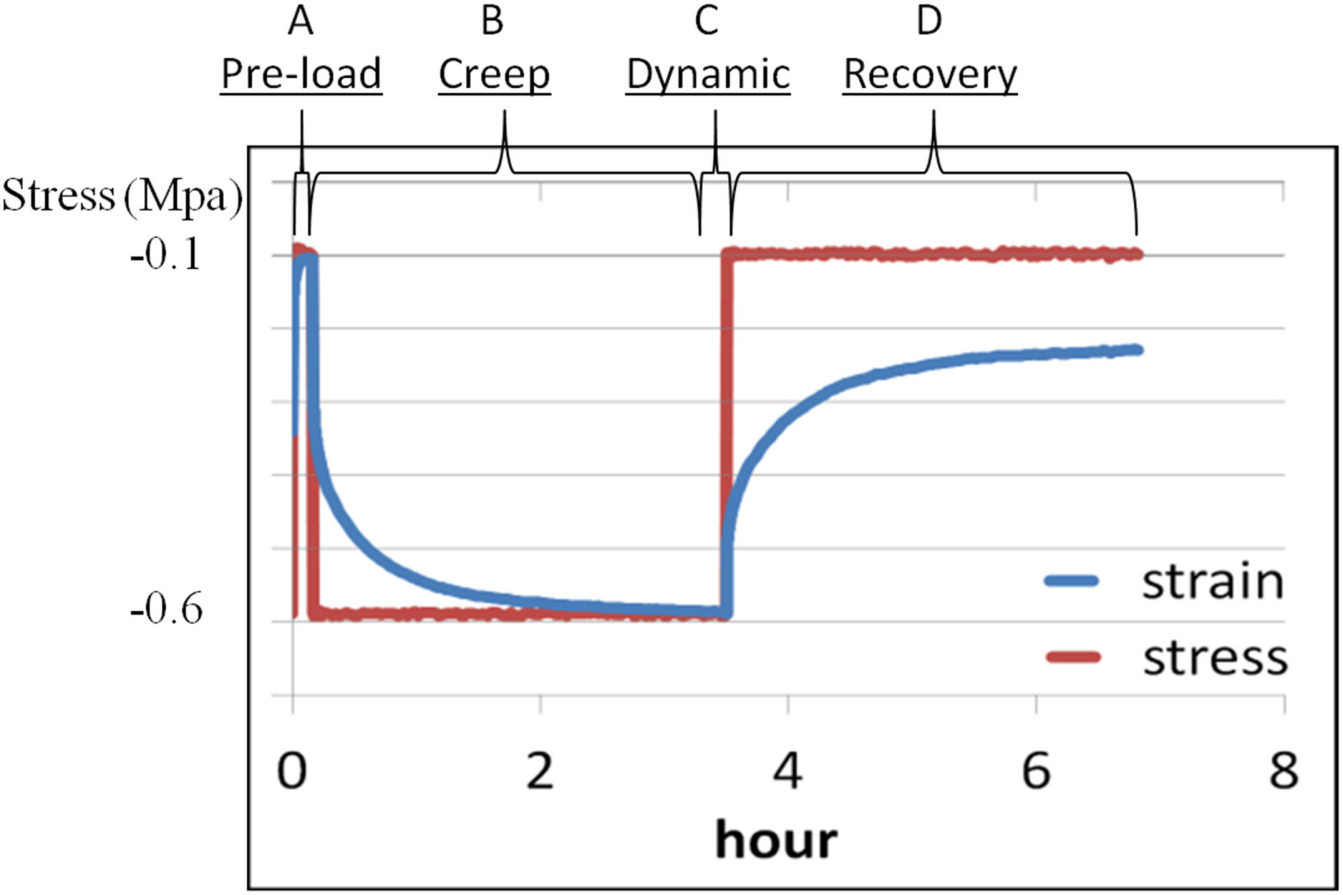
A representative stress-strain curve showing the four stage-loading protocol used for the native discs and biphasic constructs. Samples were subjected to: pre-load (A) at 0.6 MPa for 150 sec and then 0.1 MPa for 600 sec; creep (B) at 0.6 MPa for 12,000 sec; dynamic load (C) at sine stresses between 0.3 and 0.9 MPa at 0.1, 0.32, 1, 3.2 and 10 Hz (linear log scale); and recovery (D) at 0.1 MPa for 12,000 sec.

The biphasic scaffold and the disc were studied by unconfined compressive creep, dynamic mechanical analysis (DMA) and recovery (creep under reduced stress), using a bioreactor (ElectroForce 5210, BioDynamic System, Bose, Minnesota, USA) with 0.04 mm porous platens. Samples were immersed in PBS at room temperature overnight prior to the test, and then at 37°C throughout the test. A testing protocol was modified from two previous reports studying native IVD tissues [19,20]. The test in the current study was load-controlled and consisted of four stages, as shown in Fig 2. These are as follows: (A) Pre-load: ramping to and dwelling at 0.6 MPa for 150 sec, then ramping to and dwelling at 0.1 MPa for 600 sec. (B) Creep: ramping to and dwelling at 0.6 MPa for 12,000 sec. (C) Dynamic load: applying sinusoidal stress between 0.3 and 0.9 MPa at 0.1, 0.32, 1, 3.2 and 10 Hz (i.e., a linear log scale). The amplitude of loading used in the DMA corresponded to the normal range measured during daily activities (i.e., between -0.3 and -0.9 MPa) [18]. (D) Recovery: ramping to and dwelling at 0.1 MPa for 12,000 sec. The rate of ramp in Stages A, B and D were 10 N/s. In addition, the data acquisition rate was 20 Hz for the first 300 sec in Stage A, when the displacement changed rapidly, and then 1 Hz for Stages B and D. The acquisition rate was 500 Hz for Stage C, which is the default rate used by the machine software. For Stages B and D, the load-displacement data were split into two parts according to the load change, and fitted to model equations for the least square error using Excel Solver (Microsoft, Washington, USA). Thus, data describing a ramp section with ramped load within the first two sec of a stage, was fitted to Eq 1 for elastic compliance (E, in mm/N), while the remaining data, with steady load, was fitted to Eq 2 for viscous compliance (V, in mm/N), time constant (T, in s) and stretch constant (B, dimensionless), where t is time (in seconds), s(t) is displacement (in mm) at time t and F(t) is load (N) at time t.

Ex:  s(t)− s(0)= F(t) ×E          (0≤t<2) (1)

$$\text{Ex}:\text{ s}\left(\text{t}\right)-\text{ s}\left(2\right)=\text{F}\left(\text{t}\right)\;\times \text{V }\times \left(1-{\text{e}}^{-{\left(\frac{\text{t}-2}{\text{T}}\right)}^{\text{B}}}\right)\;\left(2<t\right)\;$$ (2)

With regards to Stage C, the WinTest DMA software (Bose) was used for data acquisition and analysis. Dynamic stiffness (K*) and the damping factor (tan delta) from 0.1 Hz to 10 Hz loading frequencies were obtained. Slopes of K* and tan delta against log frequency were obtained during measurement of the association between the DMA parameters and the loading frequency.

### Histology and Immunohistology

After biomechanical characterization, scaffold samples were bisected along the vertical axis and fixed with paraformaldehyde (30525-89-4, Merck, Hohenbrunn, Germany) at 4% (w/v) in PBS at 4°C overnight. After washing with PBS to remove any unreacted fixative, samples were incubated in sucrose (S5016, Sigma) at 30% (w/v) overnight, after which they were embedded in optimal cutting temperature compound (OCT) at -30°C for cryosectioning. For histology, cryosections of 15 μm were stained with Alcian blue to label GAGs. For immunohistology, 15 μm sections were incubated with H_2_O_2_ (H47055-4I, Oriental Chemicals & Lab. Supplies Ltd., Tsuen Wan, Hong Kong) at 3% (v/v) for 15 min to remove endogenous peroxidase activity, followed by pepsin (at 5% (w/v); P7012, Sigma) at 37°C to expose epitopes. The sections were then treated with blocking solution (i.e., bovine serum albumin; A4378, Sigma; at 1% (w/v)) for 20 min, after which they were incubated with a mouse anti-type I collagen primary antibody (C2456, Sigma; used at 1:2,000 dilution) at 4°C overnight, and then a horse radish peroxidase-tagged anti-mouse IgG secondary antibody (SC2005, SantaCruz, Texas, USA; at a dilution of 1:800) for 1 h. Sections were then incubated with Vectastain ABC kit reagent (PK4000, Vector Labs, CA, USA) for 30 min, followed by 3,3'-diaminobenzidine (K346811, Dako, CA, USA) for 5 min, after which they were dehydrated through an increasing concentration gradient of ethanol and xylene, and mounted under DePex mounting medium. These sections were observed using light microscopy.

### SEM characterization

After mechanical characterization, the bisected scaffold samples were fixed with glutaraldehyde (G6257, Sigma) at 2.5% (v/v) in PBS at 4°C overnight. After washing with PBS, the samples were dehydrated using an increasing concentration gradient of ethanol and then subjected to critical point drying. Dehydrated samples were fractured, mounted and sputtered coated with gold, and then observed with a Hitachi S4800 FEG scanning electron microscope.

### Isolation of rabbit IVD for comparative studies with native discs

All protocols involving animals were approved by the Committee for the Use of Live Animals in Teaching and Research of the University of Hong Kong. Two New Zealand white rabbits (> 6 months old and ~ 3.5 kg in weight) were used. Three lumbar discs (two L1-2 and one L2-3), with half of the adjacent vertebral bones, were isolated and frozen at -60°C. Before mechanical characterization, the bones adjacent to the disc were polished with sandpaper to produce smooth and parallel surfaces for fixation purpose. The discs were then soaked in PBS until the end of the mechanical test.

### Statistical Analysis

Quantitative data including the dimensions and mechanical properties were presented as the mean ± 2 standard errors (SE). The dimensions of the native disc and biphasic scaffolds with the various layers and at different stages of the test were analyzed and compared by two-way ANOVA. Compliances (E and V), the time constant (T) and the stretch constant (B) obtained from the creep and recovery tests, as well as the dynamic stiffness (K*) and damping factor (tan delta) obtained from the DMA were analyzed by one-way ANOVA with appropriate post-hoc tests. Simple linear regression analysis was performed to evaluate the linear trends during the dimension and biomechanics analyses on the biphasic scaffolds with the different numbers of AF lamellae. All the data analyses were performed with SPSS 19.0, and the significance level was set at 0.05.

## Results

### Characterization of the biphasic scaffold

**Fig 3:**
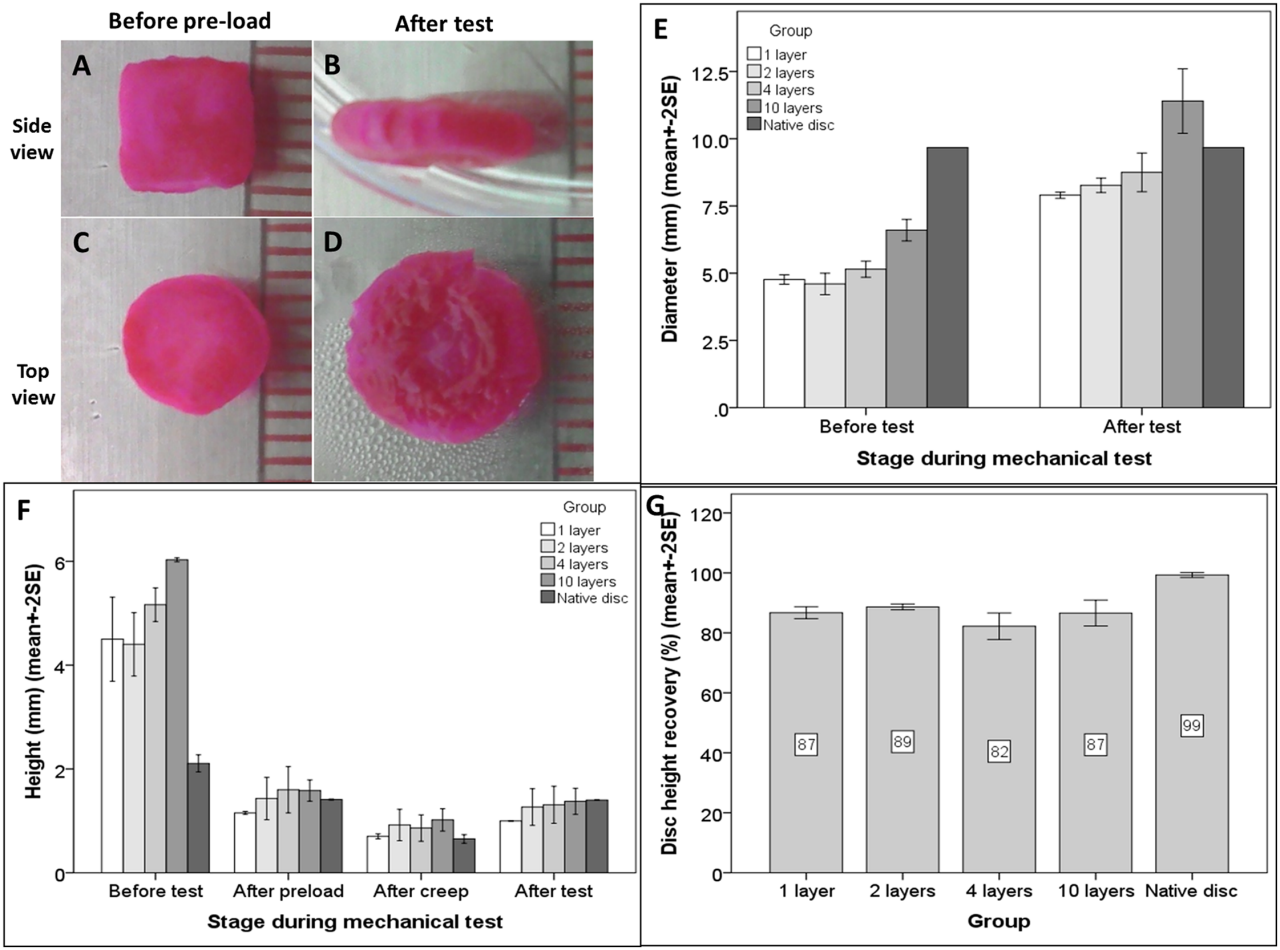
Gross appearance and dimension analysis of the samples in the different groups before and after biomechanical testing. (A-D) Gross appearance of the biphasic scaffolds from side (A-B) and top (C-D) views before (A,C) and after (B,D) the mechanical tests. (E-G) Dimension analysis of the biphasic scaffolds (with 1, 2, 4 and 10 layers of collagen lamellae) and the native disc controls at different stages during the mechanical test (i.e., before and after pre-load, after the creep, and after the test). Bar graphs show: (E) disc diameter, (F) disc height, and (G) percentage of disc height recovery. Data are expressed as mean+-2SE of n = 2–4 experiments.

The biphasic IVD scaffolds are cylindrical in shape before they undergo the pre-load steo before mechanical testing and they are disc-shaped after the tests (Fig 3A–3D). The pink color of the scaffolds corresponds to the presence of the photosensitizer, rose Bengal. Fig 3E shows the changes in diameter of the various sample groups. Two-way ANOVA indicated that there was a significant change in the diameter for each group, before pre-load and after the mechanical test (at p<0.001) and among all the groups, including the scaffolds of different layers and the native discs (at p<0.001). In addition, Bonferroni’s post-hoc tests demonstrated that the native discs exhibited significant differences when compared with the biphasic scaffolds containing 1, 2 and 4 AF-like layers (at p< = 0.001) but not with scaffolds comprised of 10 layers (p = 0.149). Furthermore, the diameter of the biphasic scaffolds significantly and linearly increased as the number of layers increased, both before pre-load (p<0.001, R^2^ = 0.856) and after the mechanical tests (p<0.001, R^2^ = 0.862). Fig 3F shows the changes in height of the disc samples. Two-way ANOVA showed that there was significant change in height among the different stages during the mechanical test (at p<0.001) and among all groups including the scaffolds with the various numbers of layers and the native discs (p = 0.045), although the latter was really marginal. Dunnett’s test showed that the disc height appeared to be significantly different before and after the pre-load as well as the creep test (p<0.001) but when we allowed for recovery, the height of the disc was not actually significantly different (p = 0.123), suggesting that it was restored. Dunnett’s test also showed that, when compared with the native discs, all the biphasic scaffolds with the different AF layers showed no significant difference in disc heights (p< = 0.267). Fig 3G shows the mean percentage disc height recovery after the mechanical test. All the biphasic scaffolds independent of the number of AF layers used, recovered to somewhere in the range of 82 to 89% of the original disc height after pre-load, while the native discs showed 99% recovery. Although the practical difference in the percentage recovery of the disc height between the biphasic scaffolds and the native disc was only ~10%, one-way ANOVA still showed statistically significant differences among groups (at p<0.001), and the difference between the native disc and all the biphasic scaffolds was significant (at p< = 0.003), independent on how many AF layers there were.

### Histology, Immunohistology and Ultrastructural analysis

**Fig 4:**
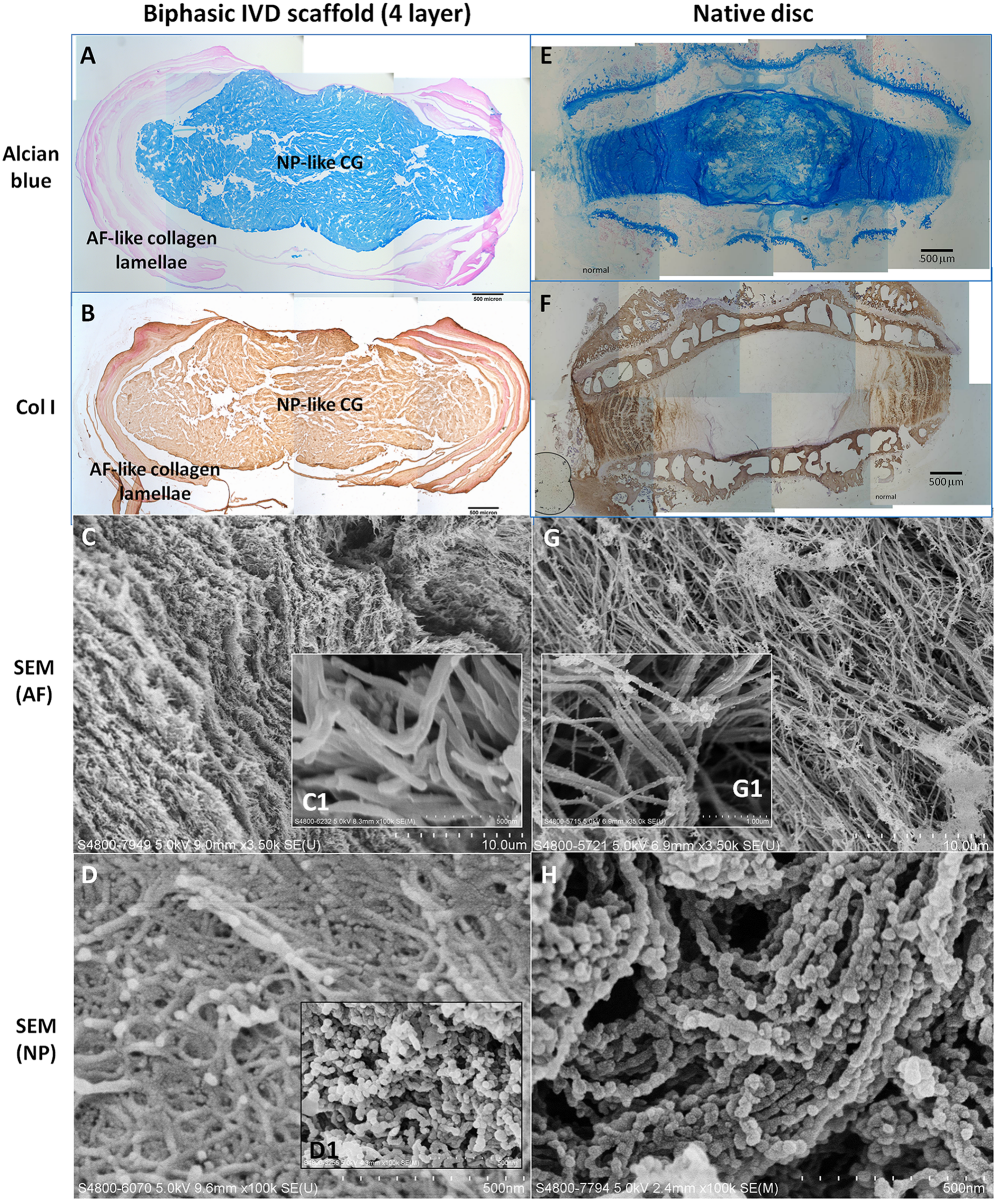
Histomorphometric and ultrastructural characterization of the fabricated biphasic scaffold and native disc. (A-D) Fabricated biphasic scaffold and (E-H) native disc; (A&E) Alcian blue staining and (B&F) immunohistochemistry of type I collagen. (C-D & G&H) SEM images. (C) AF-like collagen lamellae; (D) NP-like core with compaction; (D1) NP-like core without compaction; (G) AF lamellae in the native disc; (H) NP core in the native disc. Scale bars are 500 μm (for A-B & E-F); 10 μm (for C&G); 1 μm (for G1) and 500 nm (for D-D1&H).

Following the mechanical tests, the fabricated biphasic scaffold and native IVD constructs were sectioned and stained with either Alcian blue or an antibody against type I collagen. The histological and ultrastructure appearance of a biphasic scaffold comprised of 4 layers of AF-like collagen lamellae as shown in Fig 4A–4D, while the same characterization in native discs is shown in Fig 4E–4H. Whereas the biphasic scaffolds did not have the endplate-vertebrae structure of the native discs (Fig 4E and 4F), the dimensions and structure of both were comparable, except that the AF region in the biphasic scaffolds was thinner, being comprised of just 4 layers of collagen lamellae (Fig 4A and 4B), when compared with the 15–40 layers in native discs (Fig 4E and 4F). In the biphasic IVD scaffold, the GAG-rich NP scaffold was surrounded by an AF-like multi-lamellae structure, with a clear boundary. The NP scaffold was intensely stained with Alcian blue, indicating the successful retention of GAGs within the collagen fibrous meshwork after both the fabrication process and the mechanical test. On the other hand, the AF scaffold was not stained with the dye, and appeared pink due to the photosensitizer (Fig 4A). Immunohistology of type I collagen showed positive staining throughout the fabricated constructs (Fig 4B). In comparison, the native disc showed a typical GAG-rich NP core surrounded by multiple AF lamellae (Fig 4E and 4F), while type I collagen was mainly confined to the AF region (Fig 4F). Ultrastructural analysis of the fabricated scaffolds demonstrated that the AF region consisted of multiple interconnected lamellae structures when viewed at low magnification (Fig 4C). At higher magnification (see insert, Fig 4C1), these structures were resolved as randomly oriented, nano-sized collagen fibers. On the other hand, the AF lamellae in the native disc consisted of well-organized fibers (Fig 4G) with nano-sized fibrils (Fig 4G1), which are of comparable dimension to those found in the AF region of the biphasic scaffolds (compare Fig 4G1 with Fig 4C1). Ultrastructural analysis of the NP core in the fabricated scaffold showed thick and aggregated fibrous collagen structures intercalating with bead-like structures, which correspond to the co-precipitated GAGs (Fig 4D). For comparison, the insert (Fig 4D1) clearly shows the bead-like GAG structures in samples prior to the compaction procedure during fabrication. The NP region of the native disc showed well organized strings of fibrils covered with numerous bead-like GAG structures (Fig 4H), which were also present in the collagen-GAG NP-like core of the biphasic structure (Fig 4D1).

### Mechanical properties during creep and recovery phases

**Fig 5:**
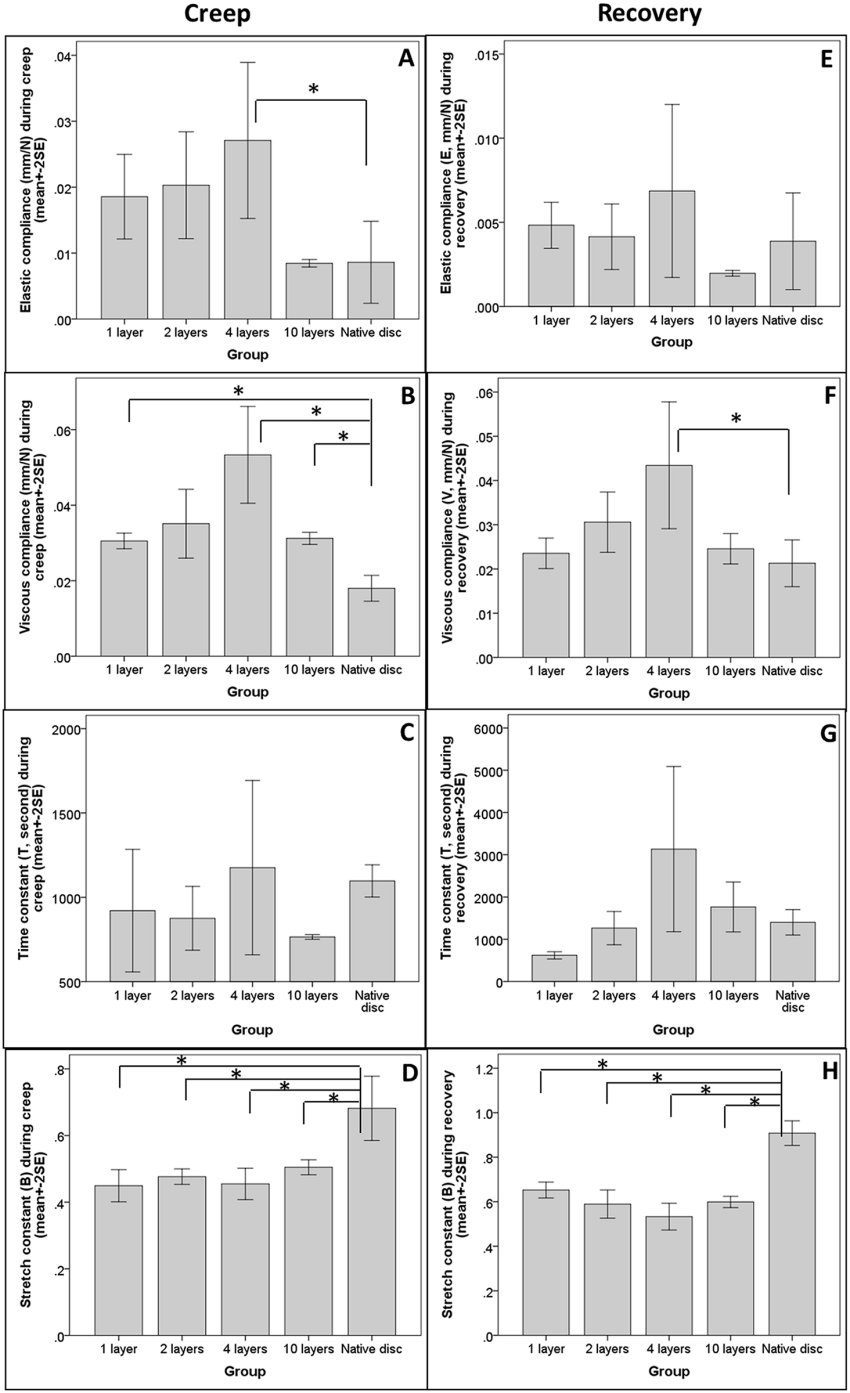
Bar charts showing the mechanical properties of samples during the creep and recovery phases of the mechanical tests. The mechanical properties of the fabricated biphasic scaffolds and native IVD (A-D) during the creep and (E-H) recovery phases. (A&E) Elastic compliance (E, mm/N); (B&F) Viscous compliance (V, mm/N); (C&G) Time constants (T, seconds) and (D&H) Stretch constants (B). Data are expressed as mean±2SE of n = 2–4 experiments.

Fig 5A shows changes in elastic compliance (E, in mm/N) during creep. Biphasic scaffolds with 1, 2 and 4 layers of lamellae seemed to have higher compliance values than did those with 10 layers and the native disc, which have similar elastic compliance. However, one-way ANOVA indicated that the difference was marginal (p = 0.067). Nevertheless, Dunnett’s post-hoc test showed that there was significant difference between the native disc and the 4-layer scaffold groups (p = 0.040) but not with the other groups including the 10-layer group (p> = 0.281). Fig 5B shows changes in viscous compliance (V, in mm/N) during creep. One-way ANOVA showed that there was significant difference in V among the different samples (p = 0.002) while Dunnett’s T3 post-hoc test showed that the differences between the native disc and all but the 2-layer groups were statistically significant (at p< = 0.049). Fig 5C shows changes in the time constant (T, in sec) during creep. One-way ANOVA showed that there was no significant difference among any of the groups (p = 0.571). Fig 5D shows the stretch constant (B) during creep. One-way ANOVA showed that there was significant difference in B among the different groups (p = 0.001), while Bonferroni’s post-hoc test showed that native disc was significantly different from all the fabricated biphasic scaffolds (at p< = 0.031). Linear regression analyses demonstrated that there was no significant linear trend in the mechanical properties as the number of layers increased (p>0.05). Instead, the association between the compliance parameters and the number of layers in the biphasic scaffold was in general non-linear, such that the compliance parameters of 1, 2 and 4 layers were higher than the 10-layer group, which was similar to that of the native disc. Fig 5E shows the changes in elastic compliance (E) during recovery; one-way ANOVA showed that the difference in E among the different groups was insignificant (p = 0.493). Fig 5F shows the changes in viscous compliance (V) during recovery; in this case, one-way ANOVA showed that there was a significant difference in V among the different samples (p = 0.040), while Dunnett’s test showed that the difference between the native disc and the 4-layer group was statistically significant (p = 0.024). Fig 5G shows the changes in the time constant (T) during recovery; here, one-way ANOVA showed that the difference was not statistically significant (p = 0.099). Fig 5H shows changes in the stretch constant (B) during recovery; one-way ANOVA showed that the difference was statistically significant (at p<0.001) while Bonferroni’s post-hoc test indicated that native disc was significantly different from all the fabricated biphasic scaffolds (at p< = 0.001). Linear regression analyses showed that there was no significant linear trend in the mechanical properties as the number of layers increased (p>0.05). Instead, the association between the number of layers of the biphasic scaffold and the mechanical parameters was in general non-linear. Raw data on the dimension and mechanical parameters of the constructs during the mechanical tests were included as Supplementary Information.

### Dynamic Mechanical Analysis (DMA)

**Fig 6:**
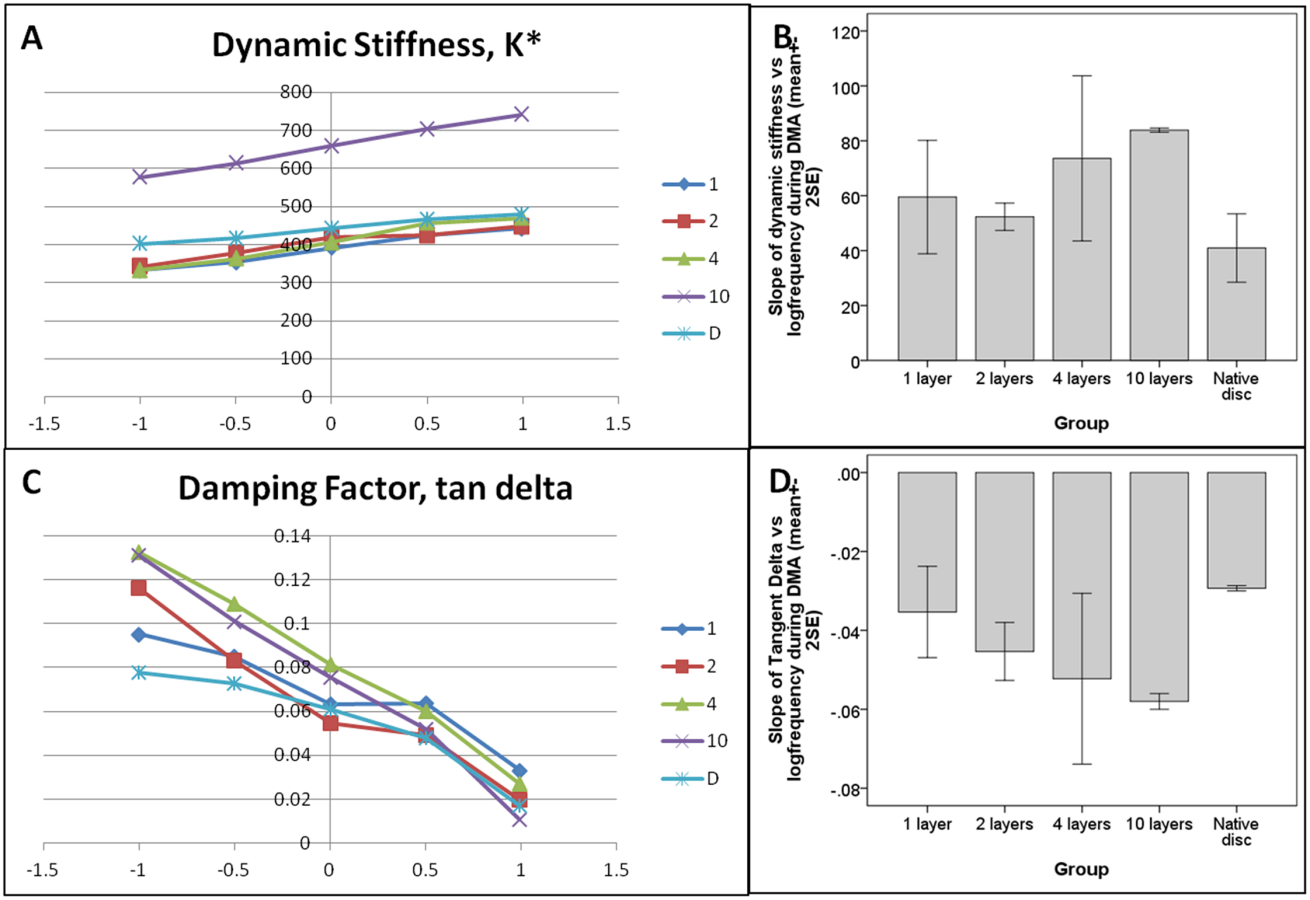
Dynamic stiffness and damping factor of samples during the dynamic mechanical analysis (DMA). (A) Line chart of the dynamic stiffness against the log loading frequency. (B) Bar chart showing the slope values measured for the dynamic stiffness-log loading frequency curves (mean+-2SE, n = 2–4). (C) Line charts of the damping factor (tangent delta) against log loading frequency; D: Bar chart showing the slopes of the tangent delta-log loading frequency curves (mean+-2SE, n = 2–4).

Fig 6 shows the changes in dynamic stiffness (K*) and the damping factor (tan delta) of samples at different loading frequencies using a log scale during DMA. Linear regression analyses showed that apart from a few exceptions in the 1-layer and 2-layer groups, all the groups showed a significant linear relationship (Fig 6A) between the dynamic stiffness and log loading frequency (at p< = 0.032). Although the overall values measured for the 10-layer group were higher than other groups, the slopes of the K*-log frequency curves (Fig 6B) were similar among all the groups including the native disc (one-way ANOVA, p = 0.149). Similarly, linear regression analyses showed that apart from a few exceptions in the 1-layer group, all the groups showed a significant linear relationship (Fig 6C) between the damping factor and log loading frequency (at p< = 0.05). The slopes of the tangent delta-log frequency curves (Fig 6D) were not significantly different among different groups (one-way ANOVA, p = 0.133) although Dunnett’s post-hoc test showed a significant difference between the native disc and the 10-layer group (p = 0.031).

## Discussion

### Structural similarities and differences between the fabricated biphasic scaffold and the native disc

The current biphasic scaffold was designed to mimic the structure of the matrix in a native disc such that it consisted of both NP and AF components. The NP-like component was an isotopic GAG-rich collageneous structure that simulated the NP matrix of a native disc in terms of its high GAG/HYP ratio and similar ultra-structural properties, as demonstrated previously [16]. Alcian blue staining of the biphasic scaffold showed the retention of GAGs in the NP-like core even after the fabrication process and the mechanical tests, which involved repeated washing and dehydration as well as compression and recovery. In the biphasic design, the NP-like core was laminated with multiple layers of photochemically crosslinked collagen gel. Nevertheless, such a gel (even one that is photochemically crosslinked), is still a hydrogel, comprising of >85% water [21] and a loose and open fibrous collagen meshwork [22]. Additional compaction procedures, including controlled dehydration via rolling on a water-absorbing filter membrane, and mechanical pre-loading via compression with a bioreactor were used to further condense the collagen hydrogel into a lamellae-like structure with a dense collagen meshwork. Before pre-loading, the construct had a greater height and smaller diameter than the native discs, but after unconfined pre-loading with free lateral deformation, the height of the sample decreased and the diameter increased to more closely imitate the dimensions of a native disc. As a result, the AF-like component simulated the AF matrix of a native disc in terms of the thin membrane-like structure and dense nanofibrous collagen meshwork. Preliminary studies on photochemically crosslinked collagen membranes provide hope that tensile properties may be similar to that of annulus fibrosus [15, 21] but it was not tested using the current fabrication method. The current scaffold design by no means reproduced the exact structure of the native disc, as it exhibited several major differences that deserve future optimization. Firstly, in the native AF, the lamellae are packed and interconnected by elastin fibres, and possesses radial transition of structures and properties towards the native NP. Our histological analysis showed that the collagen lamellae in the construct were loosely attached to each other. This might affect the mechanical performance of the construct as the lamellae could slip upon loading, especially during multiple laminations. This problem is likely to be resolved when cells are seeded in the constructs, as they will remodel the scaffolds and stimulate the photochemically-crosslinked collagen lamellae to become more inter-connected, as demonstrated previously in a subcutaneous implantation model [15]. Secondly, the AF-like component doesn’t have the same type of angle-ply structure that is exhibited by the native AF. Such a structure has previously been achieved using electrospun polymer fibers with alignment [11–13] and has shown to be important in achieving the functional properties of AF constructs [11]. In our design, we aimed to fabricate effective dense collagen lamellae before stimulating the functional remodeling of seeded cells. We achieved this by using physiologically relevant mechanical loading, including compression and torsion, which has previously been shown to promote the preferred alignment of human mesenchymal stem cells in our collagen encapsulation system [23–25]. Thirdly, the NP-like component of the fabricated biphasic scaffold contained type I collagen. Even though this was chemically modified by amination [16], it is different from the type II collagen that is present in native discs. We chose to use type I rather than type II collagen due to its abundance and superior gelation properties. In addition, we have previously shown that many cells, including NP cells [26,27], and chondrocytes [28], as well as chondrogenically differentiating [29,30] and osteogenically differentiating [31] mesenchymal stem cells, are able to remodel a template comprised of a type I collagen meshwork by synthesizing extracellular matrix, whereby one of the components is type II collagen. We therefore expect that when cells are introduced into NP-like components, they will be able to remodel the template type I collagen into a type II collagen-rich matrix in a similar way to that demonstrated for rabbit NP cells in our previous studies [26,27].

### Similarities and differences in the mechanical properties between the fabricated biphasic scaffold and native disc

An ideal disc scaffold is characterized by its ability to recover to its own height over repeated diurnal cycles of physiological loading in human discs but most bioengineered designs so far failed to mimic this mechanical function of the native disc. We showed that all the fabricated biphasic constructs, irrespective of the number of lamellae used, recovered to between 82–89% of their original height, which is approaching the 99% recovery achieved by native discs but still statistically significantly. As the recovery in disc height was independent of the number of the AF lamellae, this indicates that the recovery process is mainly influenced by the fluid replacement function of the NP-component rather than the elastic collagen meshwork rebound of the AF component. This is indeed a special feature of the mechanical function of the native disc while further optimization is necessary to achieve full recovery of lost disc height upon loading. Secondly, the elastic compliance represents the slope of force-displacement curve during the initial stage of creep or recovery, and is the elastic contribution of the solid collagen meshwork upon loading. Upon creep, all but the 4-layer scaffold showed similar elastic compliance to that of the native disc. This is due to the fact that in scaffolds with 1 and 2 layers, the pre-loading procedure over-compacts the scaffolds, leading to a lower elastic compliance of the elastic collagen meshwork than it does in scaffolds comprised of 4 layers. While this compaction effect is less prominent in scaffolds of 4 and 10 layers, the layering effect takes over after 4 layers. Thus, with 10 cycles of photochemical crosslinking in the 10-layer constructs, the elastic compliance is lower than in scaffolds with 4 layers, and hence they again exhibit a similar compliance to that of native disc. Upon recovery, the elastic compliance of all the biphasic scaffolds was similar to that of the native disc. Thirdly, viscous compliance represents the magnitude of time-dependent deformation after the stress was changed from -0.1 to -0.6 MPa in compression, and from -0.6 to -0.1 MPa in recovery. This magnitude refers to the final equilibrium state and is not related to either the time or deformation pattern. The viscous compliance was proposed to be related to the volume of fluid being displaced out of a disc by compression [19]. All the biphasic scaffolds (independent of the number of layers used) showed significantly higher viscous compliance and hence more fluid displacement, than the native disc during creep. This might be improved by further increasing the number of AF-like layers. Upon recovery, all but the 4-layer group showed similar viscous compliance and hence fluid re-absorption, when compared with that in the native disc. This is likely to be due to the over-compaction effect of the lower AF-number groups and the layering effects of the 10-layer group. Finally, the stretch constant describes the pattern of the time dependent strain, which might correspond to the path of fluid being removed from and absorbed into the disc [19]. A stretch value of 1 represents a normal exponential growth curve of strain (in which the same portion of the remaining strain is covered at equal time intervals), whereas a stretch value less than 1 represents a stretched strain curve (in which a larger portion of the remaining strain is covered at earlier time intervals, and the portion being covered diminishes in successive time intervals). This study showed that all the biphasic scaffolds showed lower stretch constants than that of native discs in both the creep and recovery stages, and this was independent of the number of AF layers. This difference in the path (including the source and direction) of fluid displacement, between the fabricated biphasic scaffolds and the native disc may be due to the absence of the endplate and vertebrae structures in the former. Therefore, the inclusion of a vertebrae-endplate-like subunit in the design might improve the mechanical performance of the biphasic scaffold and presents the goal of our future investigation. We demonstrated the feasibility of this idea in a recent study, where we incorporated a mesenchymal stem cell and collagen-based tri-layered osteochondral subunit that we previously developed [32], into a bioengineered spinal motion segment prototype [23].

### Dynamic mechanical performance of the fabricated biphasic scaffolds and native disc

Loading frequency-dependent changes in the mechanical properties of a spinal motion segment are important parameters in evaluating disc degeneration and emerging therapies [33]. The dynamic compression stiffness in a healthy human spinal motion segment increases in a linear manner with the log loading frequency [33,34]. This may be related to the stiffening and fluid flow effects, which stimulate increased transport of fluid and ions, and higher level of GAGs in the central NP at high dynamic loading frequency as the disc is compressed [33,35]. This frequency-dependent change in the dynamic stiffness was lost when the NP was removed by puncture injury [36,37], but was recovered when silicon polymers were injected into the disc [38]. Moreover, the frequency-dependent change in disc dynamic stiffness is associated with changes in the extracellular matrix upon protease digestion and crosslinking treatment in rat spinal motion segments [19,39], suggesting that the performance determined in the DMA is a sensitive parameter revealing changes in matrix structural and compositional changes. In the current study, all the biphasic scaffolds and native discs showed similar DMA performance, with a significant linear relationship between the dynamic stiffness and log loading frequency. The 10-layer biphasic scaffolds showed an even higher dynamic stiffness than the native discs. This may be due to the repeated cycles of en-sheathing, controlled dehydration and photochemical crosslinking, all strengthening the AF-like lamellae. During the DMA, all the biphasic scaffolds showed similar frequency-dependent damping behavior, when compared with that of the native discs, although the latter appeared to be non-linear. This difference may be due to multiple factors affecting the damping behavior of materials including the difference in geometry between the biphasic scaffolds and the native disc. In vivo, IVD are exposed to a very complicated mechanical environment. The current study only tested the compression mechanical properties upon creep and recovery, as well as the dynamic mechanical analysis. Other mechanical properties of the biphasic scaffolds under different modes of mechanical loading such as torsion, are to be investigated in the future.

## Conclusions

The fabricated biphasic scaffold reported in this study structurally mimics the native IVD with a collagen-GAG-rich NP core and a strengthened PCM AF scaffold. The repeated lamination process was compatible with the NP core such that the collagen-GAG-rich matrix was well retained within the AF-like lamellae. The different biphasic scaffolds were also shown to partially mimic the mechanical function of native disc. The disc height of all the scaffolds recovered by 82–89% following the mechanical test, although this was shown to be significantly different from the 99% recovery exhibited by the native disc. However, the mode of disc height recovery, (i.e., being NP-dependent and AF-independent) was shown to mimic the unique feature of native discs in the fluid replacement function. Biphasic scaffolds with 10 AF-like lamellae had the best overall mechanical performance among the various designs, owing to its similarity with native disc in most aspects, including elastic compliances during creep and recovery, and viscous compliance during recovery. The dynamic mechanical analysis showed that the dynamic stiffness and damping factor of all the biphasic scaffolds had a similar performance to that of the native disc. This study provides new evidence to facilitate future progress in the rationalized design and development of a biomimetic and mechanically viable biphasic scaffold for IVD tissue engineering and subsequent therapeutic applications.

## Supporting Information

S1 Table Data on dimensions and mechanical properties at different stages during the mechanical test. Dimensions before test, after pre-load, after creep test, after mechanical test (Height, percentage of height recovery, diameter, area and volume); Mechanical parameters at stage 1 creep: Elastic compliance (Ec, mm/N); Viscous compliance (Vc, mm/N); Time constants (Tc, seconds) and Stretch constants (Bc); and stage 3 recovery: Elastic compliance (Er, mm/N); Viscous compliance (Vr, mm/N); Time constants (Tr, seconds) and Stretch constants (Br).
(DOCX)
