# Supplementary material for: A Structurally and Functionally Biomimetic Biphasic Scaffold for Intervertebral Disc Tissue Engineering
Source: PLoS One. 2015 Jun 26;10(6):e0131827. doi: 10.1371/journal.pone.0131827 (PMC4482706; doi:10.1371/journal.pone.0131827)
Supplement: S1 Table — Dimensions before test, after pre-load, after creep test, after mechanical test (Height, percentage of height recovery, diameter, area and volume); Mechanical parameters at stage 1 creep: Elastic compliance (Ec, mm/N); Viscous compliance (Vc, mm/N); Time constants (Tc, seconds) and Stretch constants (Bc); and stage 3 recovery: Elastic compliance (Er, mm/N); Viscous compliance (Vr, mm/N); Time constants (Tr, seconds) and Stretch constants (Br). (DOCX) [file pone.0131827.s001.docx]

**Supplementary Information**

**Supplementary Table – Raw data on changes in dimension of the constructs and parameters of mechanical tests at different stages during the mechanical test.**

| **Height** | | |  | | | |  | | | | | | |  | | |  | | | |  |  |
| --- | --- | --- | --- | --- | --- | --- | --- | --- | --- | --- | --- | --- | --- | --- | --- | --- | --- | --- | --- | --- | --- | --- |
|  | | | Average | | | |  | | | | | | |  | | |  | | | |  |  |
| Group | | | h, post fabrication | | | | h, after preload | | | | | | | h, after creep | | | h after test | | | |  |  |
| 1 | | | 4.5 | | | | 1.153333333 | | | | | | | 0.701666667 | | | 1 | | | |  |  |
| 2 | | | 4.4 | | | | 1.431 | | | | | | | 0.921333333 | | | 1.266666667 | | | |  |  |
| 4 | | | 5.16375 | | | | 1.59925 | | | | | | | 0.86125 | | | 1.311 | | | |  |  |
| 10 | | | 6.0295 | | | | 1.5845 | | | | | | | 1.0185 | | | 1.3765 | | | |  |  |
| D | | | 2.107333333 | | | | 1.410666667 | | | | | | | 0.651666667 | | | 1.4 | | | |  |  |
|  | | | Stdev | | | |  | | | | | | |  | | |  | | | |  |  |
| Group | | | h, post fabrication | | | | h, after preload | | | | | | | h, after creep | | | h after test | | | |  |  |
| 1 | | | 0.7 | | | | 0.024906492 | | | | | | | 0.040698075 | | | 0 | | | |  |  |
| 2 | | | 0.529150262 | | | | 0.354374096 | | | | | | | 0.262652495 | | | 0.305505046 | | | |  |  |
| 4 | | | 0.323403128 | | | | 0.445606983 | | | | | | | 0.255476516 | | | 0.357242215 | | | |  |  |
| 10 | | | 0.026162951 | | | | 0.14495689 | | | | | | | 0.152027958 | | | 0.177483802 | | | |  |  |
| D | | | 0.142857738 | | | | 0.009291573 | | | | | | | 0.071486595 | | | 2.71948E-16 | | | |  |  |
| **%Height recovered** | |  | | |  | | |  | | | |  | | |  | | |  | |  | | |
| Group | | Average | | | | STD | | | |  |  |  |  |  |  |  |  |  |  |  |  |  |
| 1 | | 86.7318451 | | | | 1.85061301 | | | | |  |  |  |  |  |  |  |  |  |  |  |  |
| 2 | | 88.595052 | | | | 0.88186083 | | | | |  |  |  |  |  |  |  |  |  |  |  |  |
| 4 | | 82.4932684 | | | | 4.59829557 | | | | |  |  |  |  |  |  |  |  |  |  |  |  |
| 10 | | 86.8797632 | | | | 3.29774098 | | | | |  |  |  |  |  |  |  |  |  |  |  |  |
| D | | 100.123921 | | | | 1.31474132 | | | | |  |  |  |  |  |  |  |  |  |  |  |  |
| **Diameter** | | |  | | | |  | | | | | | |  | | |  |  |  |  |  |  |
|  | | | Average | | | |  | | | | | | |  | | |  |  |  |  |  |  |
| Group | | | dia, post fabrication | | | | dia, after preload | | | | | | | %dia change after test | | |  |  |  |  |  |  |
| 1 | | | 4.766666667 | | | | 7.9 | | | | | | | 165.8791778 | | |  |  |  |  |  |  |
| 2 | | | 4.6 | | | | 8.266666667 | | | | | | | 180.2424242 | | |  |  |  |  |  |  |
| 4 | | | 5.15 | | | | 8.75 | | | | | | | 169.75 | | |  |  |  |  |  |  |
| 10 | | | 6.6 | | | | 11.4 | | | | | | | 172.6102941 | | |  |  |  |  |  |  |
| D | | | 9.67 | | | | 9.67 | | | | | | | 100 | | |  |  |  |  |  |  |
|  | | | Stdev | | | |  | | | | | | |  | | |  |  |  |  |  |  |
| Group | | | dia, post fabrication | | | | dia, after preload | | | | | | | %dia change after test | | |  |  |  |  |  |  |
| 1 | | | 0.152752523 | | | | 0.1 | | | | | | | 6.98670025 | | |  |  |  |  |  |  |
| 2 | | | 0.346410162 | | | | 0.230940108 | | | | | | | 11.53554812 | | |  |  |  |  |  |  |
| 4 | | | 0.3 | | | | 0.718795288 | | | | | | | 4.787135539 | | |  |  |  |  |  |  |
| 10 | | | 0.282842712 | | | | 0.848528137 | | | | | | | 5.459280296 | | |  |  |  |  |  |  |
| D | | | 0 | | | | 0 | | | | | | | 0 | | |  |  |  |  |  |  |
| **Area and Volume** | | |  | | | |  | | | | | | |  | | |  |  |  |  |  |  |
|  | | | Average | | | |  | | | | | | |  | | |  |  |  |  |  |  |
| Group | | | area after recovery | | | | volume after preload | | | | | | | volume after recovery | | |  |  |  |  |  |  |
| 1 | | | 49.02193537 | | | | 56.52171552 | | | | | | | 49.02193537 | | |  |  |  |  |  |  |
| 2 | | | 53.70029043 | | | | 77.00139936 | | | | | | | 68.13486147 | | |  |  |  |  |  |  |
| 4 | | | 60.43638867 | | | | 93.34979462 | | | | | | | 76.74197711 | | |  |  |  |  |  |  |
| 10 | | | 102.3530887 | | | | 161.0771837 | | | | | | | 139.5406235 | | |  |  |  |  |  |  |
| D | | | 73.44171832 | | | | 103.601784 | | | | | | | 102.8184056 | | |  |  |  |  |  |  |
|  | | | Stdev | | | |  | | | | | | |  | | |  |  |  |  |  |  |
| Group | | | area after recovery | | | | volume after preload | | | | | | | volume after recovery | | |  |  |  |  |  |  |
| 1 | | | 1.240937383 | | | | 0.811259548 | | | | | | | 1.240937383 | | |  |  |  |  |  |  |
| 2 | | | 2.974630957 | | | | 20.88969671 | | | | | | | 17.9505315 | | |  |  |  |  |  |  |
| 4 | | | 10.22511193 | | | | 14.2711106 | | | | | | | 11.61201263 | | |  |  |  |  |  |  |
| 10 | | | 15.19465965 | | | | 9.239152785 | | | | | | | 2.749433677 | | |  |  |  |  |  |  |
| D | | | 0 | | | | 0.682389105 | | | | | | | 0 | | |  |  |  |  |  |  |
| **Stage 1 Creep** |  | | |  | | | | |  | | | |  | | |  | | |  | | |  |
|  | Average | | |  | | | | |  | | | |  | | |  | | |  | | |  |
| Group | Ec | | | Rsqc1 | | | | | Vc | | | | Tc | | | Bc | | | Rsqc2 | | |  |
| 1 | 0.018552619 | | | 0.955950289 | | | | | 0.030535137 | | | | 920.9250822 | | | 0.449167388 | | | 0.998911979 | | |  |
| 2 | 0.020298353 | | | 0.959061724 | | | | | 0.035099218 | | | | 875.5817783 | | | 0.476458372 | | | 0.998990225 | | |  |
| 4 | 0.027089566 | | | 0.975690439 | | | | | 0.05331306 | | | | 1175.697557 | | | 0.454733049 | | | 0.999376325 | | |  |
| 10 | 0.008456276 | | | 0.993054105 | | | | | 0.03122068 | | | | 764.8918655 | | | 0.504624703 | | | 0.999315376 | | |  |
| D | 0.008611077 | | | 0.976332104 | | | | | 0.017983407 | | | | 1097.274512 | | | 0.681511252 | | | 0.999838113 | | |  |
|  | Stdev | | |  | | | | |  | | | |  | | |  | | |  | | |  |
| Group | Ec | | | Rsqc1 | | | | | Vc | | | | Tc | | | Bc | | | Rsqc2 | | |  |
| 1 | 0.005555117 | | | 0.020284094 | | | | | 0.001795516 | | | | 314.5205276 | | | 0.041734976 | | | 0.000389872 | | |  |
| 2 | 0.007018394 | | | 0.014333782 | | | | | 0.007889239 | | | | 163.889367 | | | 0.020065275 | | | 0.000494358 | | |  |
| 4 | 0.011843363 | | | 0.012853392 | | | | | 0.012821381 | | | | 516.822682 | | | 0.047102218 | | | 0.000554938 | | |  |
| 10 | 0.000401061 | | | 0.002333533 | | | | | 0.001137732 | | | | 9.838238619 | | | 0.015864001 | | | 0.000407802 | | |  |
| D | 0.005380423 | | | 0.004651409 | | | | | 0.002969995 | | | | 82.45887197 | | | 0.08343981 | | | 7.41305E-05 | | |  |
| **Stage 3 Recovery** | |  | | |  | | |  | | | |  | | |  | | |  | |  | | |
|  | | Average | | |  | | |  | | | |  | | |  | | |  | |  | | |
| Group | | Er | | | Rsqr1 | | | Vr | | | | Tr | | | Br | | | Rsqr2 | | %Height recovered | | |
| 1 | | 0.00482554 | | | 0.81083276 | | | 0.02353264 | | | | 619.900493 | | | 0.65268232 | | | 0.99141316 | | 86.7318451 | | |
| 2 | | 0.00414255 | | | 0.71933945 | | | 0.03059326 | | | | 1263.96565 | | | 0.5892635 | | | 0.99740282 | | 88.595052 | | |
| 4 | | 0.00686063 | | | 0.7350925 | | | 0.04341998 | | | | 3132.80757 | | | 0.53311023 | | | 0.9986605 | | 82.4932684 | | |
| 10 | | 0.00197294 | | | 0.9568597 | | | 0.02456367 | | | | 1763.58099 | | | 0.59894842 | | | 0.998875 | | 86.8797632 | | |
| D | | 0.0038758 | | | 0.92554722 | | | 0.02128213 | | | | 1400.1306 | | | 0.9083455 | | | 0.9986643 | | 100.123921 | | |
|  | | Stdev | | |  | | |  | | | |  | | |  | | |  | |  | | |
| Group | | Er | | | Rsqr1 | | | Vr | | | | Tr | | | Br | | | Rsqr2 | | %Height recovered | | |
| 1 | | 0.00118496 | | | 0.07991814 | | | 0.00298466 | | | | 74.4730859 | | | 0.03096513 | | | 0.00453183 | | 1.85061301 | | |
| 2 | | 0.00168744 | | | 0.10058647 | | | 0.00590383 | | | | 340.447733 | | | 0.0549286 | | | 0.00087302 | | 0.88186083 | | |
| 4 | | 0.00513669 | | | 0.15434653 | | | 0.01432665 | | | | 1953.35821 | | | 0.06011003 | | | 0.0007276 | | 4.59829557 | | |
| 10 | | 0.00012006 | | | 0.00078829 | | | 0.00243742 | | | | 417.512053 | | | 0.01794152 | | | 0.00014815 | | 3.29774098 | | |
| D | | 0.00248773 | | | 0.06147866 | | | 0.00458073 | | | | 260.997651 | | | 0.04797972 | | | 0.00060226 | | 1.31474132 | | |
